# Study protocol: Hypersexual and hyposexual behavior among adults diagnosed with alcohol- and substance use disorders—Associations between traumatic experiences and problematic sexual behavior

**DOI:** 10.3389/fpsyt.2023.1088747

**Published:** 2023-03-16

**Authors:** Dennis Jepsen, Tobias Luck, Marie Bernard, Irene Moor, Stefan Watzke

**Affiliations:** ^1^Institute of Medical Sociology, Interdisciplinary Center of Health Sciences, Medical Faculty, Martin-Luther-University Halle-Wittenberg, Halle (Saale), Germany; ^2^Faculty of Applied Social Sciences, University of Applied Sciences Erfurt, Erfurt, Germany; ^3^Department of Psychiatry, Psychotherapy, and Psychosomatics, University Hospital Halle, Halle (Saale), Germany

**Keywords:** alcohol addiction, chemsex, childhood trauma, compulsive sexual behavior, drug addiction, libido, sexual dysfunction, substance use disorder

## Abstract

**Background:**

Hypersexual and hyposexual behaviors are common concomitant of substance use disorders (SUD). On the one hand, the regular consumption of alcohol or illegal drugs can lead to hypersexual or hyposexual behavior due to its effects on the organism; on the other hand, the use of psychotropic substances is also used as a coping strategy concerning already existing sexual impairments. The aforementioned disorders show similarities in terms of their etiology, as traumatic experiences get special attention as potential risk factors for the development of addictions, hypersexual, and hyposexual behavior.

**Objectives:**

The study aims to explore the association between SUD characteristics and hypersexual/hyposexual behavior, and a potential moderating effect of early traumatic life events by answering the following research questions: (1) Do people with SUD differ from a sample of people with other psychiatric disorders regarding hypersexual and hyposexual behavior? (2) What are the associations between the presence of sexual problems and different characteristics of the SUD (e.g., mono vs. polysubstance use, type of addictive substance, intensity of the disorder)? (3) What influence do traumatic experiences in childhood and adolescence have on the existence of sexual disorders among adults with a diagnosed SUD?

**Method:**

The target group of this cross-sectional ex-post-facto study comprises adults diagnosed with an alcohol- and/or substance use disorder. Data will be collected with an online survey, which will be promoted via several support and networking services for people diagnosed with SUD. Two control groups will be surveyed, one consisting of people with other psychiatric disorders than SUD and traumatic experiences, and one healthy group. Relations between the dependent variables (hypersexual and hyposexual behavior) and independent variables (sociodemographic information, medical and psychiatric status, intensity of the prevalent SUD, traumatic experiences, and symptoms of posttraumatic stress disorder) will be initially calculated via correlations and linear regression. Risk factors will be identified via multivariate regression.

**Discussion:**

Gaining relevant knowledge promises new perspectives for prevention, diagnosis, case conception, and therapy of SUDs as well as problematic sexual behaviors. The results can provide more information about the importance of psychosexual impairments regarding the development and maintenance of SUDs.

## 1. Introduction

Because of its effect on the organism the regular consumption of drugs can strongly influence the level of sexual activity. The harmful use and addiction due to alcohol or illicit drugs can have both excitatory and inhibiting effects on sexual behavior (1). In relevant literature, three possible domains on the relation between alcohol or drug use, and sexual behavior are considered: (a) the use of substances to enhance sexual activity or arousal, (b) the relation between substance use and harmful or risky sexual behavior, and (c) sexual dysfunction as a consequence of substance consumption (2, 3). Thus, the consumption of, e.g., alcohol, cannabis, cocaine, amphetamines, or hallucinogen substances can increase sexual arousal short-termly, while the long-term use of, e.g., alcohol, opioids, or sedatives is often associated with impairments in sexual function (1). Consequently, two poles of sexual behavior can be of interest from a psychiatric perspective: hypersexual (an extraordinarily high level of sexual activity and arousal) and hyposexual behavior (an extraordinarily low level of sexual activity and arousal). At the same time, the influence of traumatic experiences in the life course plays an important role regarding the etiology of addictive disorders, as well as hypersexual and hyposexual behaviors. However, the complex linking system between prevalent SUDs, hypersexual and hyposexual behavior, as well as traumatic experiences has not been explored sufficiently, which comprises the general aim of this study. In the following, previous research on the associations between the respective factors is described.

### 1.1. Hypersexual behavior and substance-related addiction

Clinical relevant hypersexual behavior is classified as compulsive sexual behavior disorder (CSBD; 6C72) in the ICD-11 (4). It is characterized by “[…] a persistent pattern of failure to control intense, repetitive sexual impulses or urges resulting in repetitive sexual behavior. Symptoms may include repetitive sexual activities becoming a central focus of the person’s life” (4). Possible consequences are the neglect of important relationships, activities, and responsibilities, as well as the resulting impairments in familial, social, professional, educational, or other important areas of life (5). The prevalence of hypersexual behavior varies between 1% and around 10%, thus these estimations should be interpreted with caution (6). Since the diagnosis of CSBD was first implemented in ICD-11 in 2022, in previous research varying definitions and scales for measuring hypersexual behavior were used, so their results are not comparable in most cases (6). Several studies investigated the associations between a prevalent SUD/substance consumption and hypersexuality. In the following presentation of the current state of related research, differing aspects of hypersexuality were examined, while it is necessary to extinct between the constructs hypersexual behavior (defining subclinical behavioral deviations) and CSBD (referring to the diagnosis according to ICD-11).

Among people with SUDs, the prevalence of hypersexual behavior is estimated at around 40% and therefore significantly higher compared to those without SUD (7). Alcohol dependence was found in about 16%, and risky use of alcohol in 44% of respondents diagnosed with CSBD (4). Furthermore, positive associations were identified between the severity of substance dependence and the intensity of hypersexual behaviors in people diagnosed with cocaine or crack dependence (8). But also a risky use, especially of alcohol, cannabis, and cocaine was associated with clinically relevant hypersexual behaviors (9). Further studies indicated that both the prevalence and the expression of hypersexual behavior as a comorbidity to dependence diseases differ according to the type of addictive substance, meaning that the risk to develop hypersexual behavior seems to be significantly higher in people with poly-drug addiction than in people with isolated dependence (8). CSBD is considered both a gateway addiction and a surrogating addiction during the treatment of SUDs (10). The targeted use of drugs to evoke specific physical and/or psychological effects during sexual activity (e.g., expanding sexual performance or achieving sexual disinhibition) also plays a role in this context (11). This behavior, defined as chemsex, is associated with sexual risk behaviors (12, 13), infection with sexually transmitted diseases (12, 14, 15), impairments in social functioning (16), the secondary development of psychosis, and the resulting perception of sex without drug influence as emotionless or non-exciting (11). Further, MSM who practice chemsex report significantly higher incidences of non-consensual sex (16).

### 1.2. Hyposexual behavior and substance-related addiction

Conversely, sexual dysfunction is also known to be a possible concomitant of SUDs as well as the abuse of alcohol and illegal substances. According to ICD-11, sexual dysfunction is classified in the chapter conditions related to sexual health (HA00 to HA03) (4, 17). A distinction is made between hypoactive sexual desire dysfunction (HA00), sexual arousal dysfunction (HA01), orgasmic dysfunction (HA02), and ejaculatory dysfunction (HA03). Corresponding diagnoses are assigned if the symptoms persist or recur over a longer period of time and there is subjective suffering due to the complaints (4). The forms of sexual dysfunction are characterized in more detail in Table 1.

**Table 1:** Forms of sexual dysfunction according to ICD-11.

Diagnosis	Indicators
HA 00: Sexual desire dysfunction	Absence/severe reduction in sexual desire/to perform sexual activities; absence of sexual fantasies, thoughts, responses to sexual stimuli; difficulty maintaining arousal during sexual activities (4).
HA01: Sexual arousal dysfunction	Absence/severe reduction of sexual arousal; *In biological females:* Absence/reduced reactive or genital arousal, absence of genital and/or non-genital sensations during sexual activity (18); *In biological males:* erectile dysfunction–difficulty achieving/maintaining an erection during sexual activity; reduction in rigidity of erection (19).
HA02: Orgasmic dysfunction	Marked delay, severe reduced frequency or absence of female orgasm, marked reduction in intensity of orgasmic experience (20).
HA03: Ejaculatory dysfunction	Latency of male orgasm; Premature ejaculation (ejaculatio praecox): Inability to delay ejaculation or ejaculation about one minute of active penetration (21); Delayed ejaculation (ejaculatio retarda): Lack of ability to achieve ejaculation or increased latency of ejaculation with sufficient stimulation and present desire to ejaculate (22).

It is important to distinguish between sexual disorders (which require treatment) and subclinical sexual functioning problems (23). These subclinical sexual functioning problems (also called hyposexual behaviors) include individual symptoms of the disorders described above, without completely fulfilling the diagnosis (e.g., if there is no psychological strain regarding the sexual activity). However, individual complaints, e.g., with regard to one’s own body image (e.g., due to severe penile curvature or general dissatisfaction with one’s own body) or sexual performance (e.g., fear of failure, sexual inhibitions) can also lead to insecurities and limited sexual activity (24).

Sexual dysfunction can persist long after abstinence from alcohol or drugs. Possible reasons for that are changes in hormone balance and neuronal structures (25). Particularly prominent seems to be the association between alcohol dependence and the existence of sexual dysfunctions, since it has already been highlighted in numerous studies (26–28), with the prevalence of sexual dysfunctions ranging from around 29% (26) to 58% (29). In particular, dyspareuinia (vaginal pain during penetration) and vaginal dryness (absence of vaginal lubrication) in women (27) and erectile dysfunction, ejaculatio praecox, and reduced sexual desire in men (27–29) have been identified as comparatively common disorders in people with SUDs. Moreover, associations with sexual dysfunction have also been reported in addiction due to illegal substances, as in addiction to opioids (esp. heroin, methadone, and buprenophine) (27, 30), crack (37.2%), cocaine (20.6%), and marijuana (6.3%) (26). In addition, a study by Dolatshahi et al. (31) could identify associations between methamphetamine abuse and sexual dysfunction in men. Thus, single use of methamphetamines appears to lead to increased duration, quantity, and subjectively perceived quality of sexual activity. However, with increasing time or repetition of use, associations with decreased libido, erectile dysfunction, and ejaculatio praecox become conspicuous, with consumption-induced cognitive changes as possible explanations (31).

### 1.3. Relevance of traumatic experiences

The influence of traumatic experiences in the life course plays an important role regarding the etiology of addictive disorders, as well as hypersexual and hyposexual behaviors. In this context, traumatic experiences represent a risk factor for the development of addictive disorders (32), as well as hypersexual and hyposexual behaviors (33). In particular, associated dysfunctional family structures, breaches of trust with important attachment figures, and experiences of violence have been identified as important risk factors (34). Trauma in childhood and adolescence was reported by up to 70% of people with substance-related addiction (32). In addition, the diagnoses of substance use disorder and disorders specifically associated with stress often occur simultaneously (25), and addiction disorders may develop as a consequence of prevalent post-traumatic stress disorder (PTSD), especially when substance use is utilized as a strategy to cope with the symptoms associated with PTSD (35). Trauma in childhood and adolescence is also associated with an increased likelihood of relapse into alcohol addiction (36).

Moreover, hypersexual and hyposexual behavior are considered frequent psychosocial consequences of experienced violence in the relevant literature (34, 37, 38). A distinction is made between sexual-related consequences of sexual and non-sexual traumatic experiences (39). Sexual dysfunctions in combination with intrusive and/or dissociative experiences as well as accompanying psychological and/or physical stress reactions during sexual activities are particularly common (hence the term sexual PTSD is used in relevant literature) (40). Furthermore, hypersexual behavior is described as a possible post-traumatic stress reaction, which can express by reduced bonding ability and reduced self-reduction as well as (resulting) sexual risk behavior (41). However, the linking between traumatic experiences and behavioral addictions, such as CSBD, is considered under-researched. The few available studies, however, focused on other specific target groups than in this study. For example, the results of a study by Davis and Knight (42) were able to show an association between traumatic experiences in childhood and adolescence and the presence of hypersexual behavior in male sexual offenders. In another study by Castellini et al. (43), a corresponding association was found in female students but not in male students.

### 1.4. Theoretical bundling and deduction of objectives

**Figure 1:**
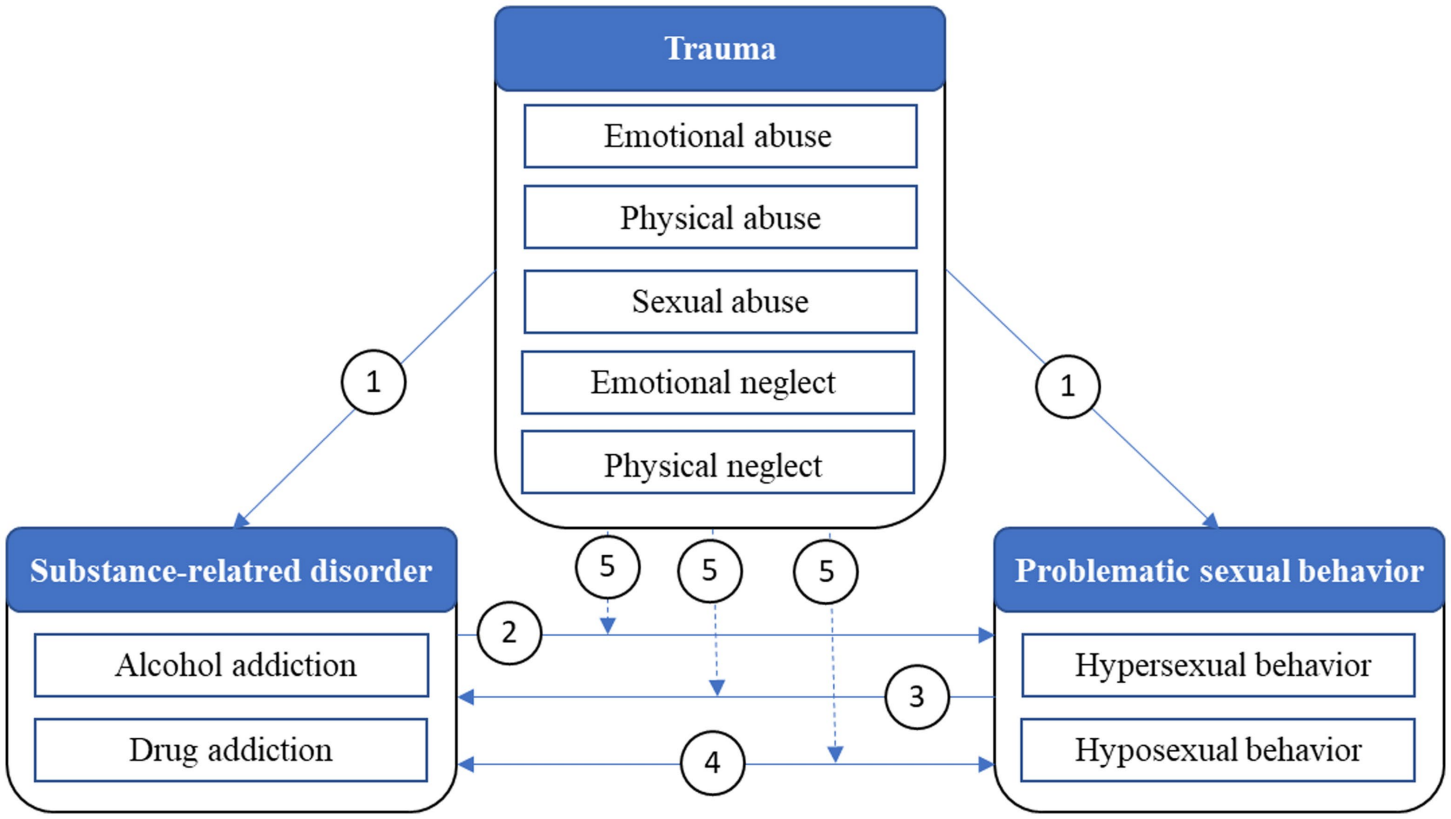
Possible relations between substance-related disorders, problematic sexual behaviors, and traumatic experiences.

In Figure 1, possible relations between substance-related disorders, hypersexual and hyposexual behavior, and traumatic experiences according to relevant literature are shown. As described above, traumatic experiences in childhood and adolescence are a possible risk factor for the development of SUDs as well as problematic sexual behavior patterns (see arrow 1). At the same time, SUDs often seem to be accompanied by corresponding problematic sexual behaviors. Sexual changes may be the result of other symptoms of the disorder (see arrow 2), be the cause of its development and maintenance (see arrow 3), or manifest as a symptom and occur simultaneously with the disorder (e.g., due to shared risk factors) (see arrow 4) (44).

The complex linking system between existing addictive disorders and hypersexual, as well as hyposexual behavior has not been explored sufficiently, while the results available to date appear inconclusive and difficult to compare. However, Efrati and colleagues (45) investigated, *inter alia*, the relation between early-life trauma and CSBD among young women with SUD. Their results indicate that young women with SUDs have higher-rated CSBD-symptoms as the healthy controls without SUD. In addition, SUD as well as CSBD were associated with childhood emotional abuse. However, negative life events were only related to CSBD, but not with SUD. To the best of our knowledge, no other study currently exists that examines the relation between the existence of SUDs and hypersexual/hyposexual behavior, and in particular the influence of traumatizing experiences on this relation (see arrow 5). Furthermore, there is little clarity whether the postulated associations between early trauma and the development of a sexual behavioral or functional disorder are specific to individuals with substance use disorders, or whether this association is also found in individuals not affected by it. As a result of the current state of knowledge described above and the existing research gaps, the following research questions have emerged for the investigation of the issue:

1. Do people with SUDs differ from a sample of people with other psychiatric disorders regarding hypersexual and hyposexual behavior?
2. What are the associations between the presence of sexual problems and the character of the SUD (e.g., mono vs. poly substance use, type of addictive substance, intensity of the disorder)?
3. How are traumatic experiences in childhood and adolescence associated with problematic sexual behavior among adults with a diagnosed SUD?

Gaining relevant knowledge promises new perspectives for diagnosis, case conception, treatment and therapy of SUDs as well as sexual problems in a psychiatric and therapeutic context.

## 2. Methods and analysis

### 2.1. Sample and inclusion criteria

The target group of this study are adults (aged 18 and above) diagnosed with a SUD. Data will be collected via an online survey, which will be promoted via several support and networking services for people with SUD (e.g., interest groups, internet forums, online self-aid groups, and online counseling services in Germany, more information stated in the section *Procedure*). Additionally, two control groups will be surveyed. The first control group will consist of people with other psychiatric disorders than addiction and traumatic experiences. They will be contacted via self-aid groups and counseling centers. The second control group comprises people without any diagnosed psychiatric disorder, which will be recruited via social media (Instagram, Twitter, Facebook). An *a-priori* power analysis was conducted via the software G-Power. The required sample size of the group of people with SUD was calculated 146 (F-test, multiple linear regression, effect size *f*^2^ = 0.15, *α* = 0.05), whereas the required sample size of the control groups was calculated 88, respectively (*t*-test, difference between two independent means, effect size *d* = 0.5, *α* = 0.05). Hence, the total required sample size is 322.

### 2.2. Study design and questionnaire

Within a cross-sectional ex-post-facto study, the required data will be collected via an online survey tool. In Table 2, the effect variables of the research proposal are listed.

**Table 2:** Overview of dependent and independent variables.

Variable	Scales
**Dependent variables**	
Hypersexual behavior	Compulsive Sexual Behaviour Scale (CSBD-19)
Hyposexual behavior	Sexual Behavior Questionnaire (SBQ)
**Independent variables**	
Sociodemographic information age, gender assignment, current place of residence, marital status, religious affiliation, educational level/professional qualification, labour situation	Direct questioning
Medical status Presence of somatic/psychiatric diagnoses, medication, pension preservation	Direct questioning
Characteristics of alcohol/substance use disorder Related substances Intensity of alcohol addiction Intensity of drug addiction Addiction and psychiatric disorders among family members	Direct questioning Alcohol Use Disorders Identification Test (AUDIT) Drug Abuse Screening Test (DAST) Direct questioning
Traumatic experiences in childhood and youth	Child Trauma Questionnaires (CTQ)
Currently prevalent symptoms of a traumatic disorder	Impact of Event Scale (IES-R)

#### 2.2.1. Hypersexual behavior

The German version of the Compulsive Sexual Behavior Scale (CSBD-19) by Böthe et al. (46) will be used to measure clinically relevant hypersexual behavior. The scale is based on the ICD-11 diagnosis of CSBD and measures the construct via the subscales control, salience, relapse, dissatisfaction, and negative consequences using 19 items (four-pointed Likert-scales with answer options between 1 = *totally disagree* and 4 = *totally agree*) in total. The CSBD cut-off value is reached at a total sum score of ≥50 (46).

#### 2.2.2. Hyposexual behavior

Hyposexual behavior will be measured by using the German version of the Sexual Behavior Questionnaire (SBQ) (47). The SBQ assesses indications for the presence of sexual dysfunction via six items addressing sexual behavior applied to all sexes and complementary to that, gender-specific hyposexual behaviors in biological women (five items) and men (six items) on a Likert-scale. The index of sexual dysfunction will be calculated via the measured variables desire for sex, capability to sexual arousal, capability to sexual enjoyment, satisfaction with sexual life and orgasm. A higher SBQ-score indicates a more intense hyposexual behavior (47).

#### 2.2.3. Gender

Gender assignment will be measured in two steps according to the recommendations of Muschalik et al., using the items *What sex was registered at your birth certificate* and *How do you designate your gender yourself*, to shape the survey inclusive and gender-sensitive according to current scientific standards (48).

#### 2.2.4. Substance-related disorder

To get information about characteristics of the prevalent addiction disorder, the related addictive substances will be recorded via two items: *Please indicate which substances the substance use disorder is related to* and *Please indicate which of the stated substances causes you the most health problems* with a list of several substance types as answer options. The intensity of the addictive disorder will be measured in different ways depending on the type of related substance. In the case of a prevalent alcohol addiction, the German version of the Alcohol Use Disorders Identification Test (AUDIT) will be used, which measures the current intensity of an alcohol addiction via three subscales (alcohol consumption, dependence, and alcohol-related consequences) consisting of eight five-pointed and two three-pointed Likert-scaled items (49, 50). Depending on the determined score, the severity levels of the prevalent alcohol addiction can be derived, while values from 0 to 7 represents an abstinence/a low-risk use, 8 to 15 a risky use, 16 to 19 a harmful use, and 20 to 40 a remaining chronic alcoholism (50).

The Drug Abuse Screening Test (DAST-10) will be included to measure the intensity of a prevalent addiction due to illegal drugs, and consists of ten dichotomous items (answer options *yes* and *no*) (51). The determined sum score characterizes the degree of problems related to the drug abuse, while values from 1 to 5 represent a low or moderate level, 6 to 8 a substantial level, and from 9 to 10 a severe level (52). Since currently no German version of the DAST exists, the scale will be translated into German by conducting a forward-backward translation (53).

In addition, the number of hospitalizations due to the stated SUDas well as information on whether the participants are currently abstinent will be determined.

#### 2.2.5. Trauma

Using the German version of the Childhood Trauma Questionnaire by Bernstein et al. (54–56) trauma in childhood and adolescence will be assessed using 28 ordinal five-point scaled items. The questionnaire includes five subscales – emotional abuse, physical abuse, sexual abuse, emotional neglect, and physical neglect (56). Furthermore, the German version of the Impact of Event Scale (IES-R) by Weiss and Marmar will be used to identify prevalent symptoms of a PTSD (intrusion, avoidance, and hyperarousal) via 22 ordinal four-point scaled items (57).

### 2.3. Procedure

A pretest will be conducted, while at first a technical check of the survey instrument should show its functionality on web-browser and smartphone. In the second part of the pretest, professionals of the care and treatment system addressing people with substance-related disorders, including social workers, addiction therapists, and physicians specialized in addiction treatment will be invited to evaluate the survey instrument regarding its suitability for people with substance-related disorders. According to the recommendations by Lenzner et al. (58), around 20 participants should be included into the pretest, because of economic reasons and the fact that the most problems with the items can usually already be identified with this number of test persons.

Data will be collected anonymously via an online questionnaire and advertised cooperating with various support and networking services for people with addiction. After careful exploration of the field, visible services, i.e., ten websites for information and mediation to self-aid groups, eight internet forums, five umbrella associations, three self-aid apps, and two online-counseling websites, will be contacted via e-mail to inform them about the purpose of the study and will be asked to advertise it on their websites. Five umbrella associations for psychiatric care in Germany will be contacted, and asked to promote an adapted version of the survey on their websites to recruit the control group of people with other psychiatric diagnoses than substance-related addictions with traumatic experiences. Further, social media handles on Instagram, Facebook and Twitter will be created to recruit the healthy control group.

At the beginning of the survey, the participants will be informed that the questionnaire deals with sensitive issues, such as mental diseases and traumatic experiences. The participants will be advised to consider this information when deciding whether to participate in the study or not. At this point, reference is also made to low-threshold offers of help, which can be utilized if the answering of the questions should lead to psychological stress. Furthermore, information on data processing and data protection will be stated. The participants must confirm the data protection consideration and the declaration of consent to participate.

### 2.4. Biometrics and data analyses

The statistical analysis will be conducted via the software IBM Statistical Package for Social Sciences (SPSS), version 28. Data will be analyzed univariate, bivariate, and multivariate, while the presence of hypersexual or hyposexual behavior will be considered as dependent variables, and all other measured constructs as independent variables. Relations between the dependent and independent variables will be initially calculated via correlations and linear regression. Risk factors will be identified via multivariate regression. The effect of moderating or mediating variables will be controlled via particularly stratified analyses. Comparisons between the investigated groups of participants will be conducted via the appropriate inference statistical tests.

## 3. Discussion

### 3.1. Expected outcomes

The study aims firstly to provide a data basis to support the above mentioned associations between alcohol/drug addictions and problematic sexual behaviors, described in the relevant literature and only sparsely investigated in previous studies. To our best knowledge, this is the first study which investigates the association between substance-related addictions and hypersexual/hyposexual behavior focusing on the influence of traumatic experiences. Within this study, first insights addressing mediating and moderating effects of the prevalence of traumas on the relation between addiction and clinically apparent problematic sexual behaviors can be derived. Besides the statistical analysis of the topic, it will be a major objective of the study to discuss the meaning of the results for prevention and psychotherapeutic treatment.

Prevention should start before addictive patterns of alcohol and drug consumption are shaped, therefore the school context might be an important setting for prevention programs in general. Although projects addressing the prevention of developing substance use disorders are common in German schools, as well as sex education programs, the interactions between addictive and sexual behavior are not discussed sufficiently in preventive context. Thus, the practicing of chemsex and the related risks of developing drug addiction, compulsive sexual behavior, and/or sexual dysfunctions should be involved in preventive context, especially because its increasing importance in health-related sciences. Hence, appropriate sexual risk behaviors should be reflected according to the medical, psychological and social perspectives on them in an educational and preventive context.

Moreover, the crucial relevance of the patient’s individual sexuality should get more space in psychotherapeutic treatment of substance use disorders. As problems in the sexual life can lead to emotional stress, self-doubt, impairments in important areas of life and many other consequences, they can be understood as maintaining factors of psychiatric diseases in general, and addictive disorders in particular. By interpreting the results of this study, the role of sexual problems as a risk factor for the development and maintenance of substance use disorders, they should be elicited by proposing new etiological theories including aspects of sexuality.

### 3.2. Limitations

While interpreting the results it should be considered that the transferability of the results to the total population of people with addictions has limitations. For example, only those will participate in this survey who are integrated in the support system of addiction care in Germany, use online services of addiction care, and are in a stable phase of their addiction disease. Furthermore, to diagnose addictions, CSBD, or sexual dysfunctions, a personal medical or psychotherapeutic consultation is required. Within this study, the above listed scales are only able to provide indications for prevalent particular disorders and problematic behaviors among the participants and cannot be equated to diagnosis from qualified personnel. Conclusively, the ex-post-facto study design does not allow any conclusions about causal relations between substance-related disorders, problematic sexual behavior, and traumatic experiences but will deliver first insights.

## Ethics statement

The studies involving human participants were reviewed and approved by Ethical committee of the medical faculty of the Martin-Luther University Halle-Wittenberg, Germany. Written informed consent for participation was not required for this study in accordance with the national legislation and the institutional requirements.

## Author contributions

DJ is the main contributor to the study design, biometrics, and wrote the manuscript. SW and TL contributed to the study design. TL, MB, IM, and SW reviewed the study protocol. All authors contributed to the article and approved the submitted version.

## Conflict of interest

The authors declare that the research was conducted in the absence of any commercial or financial relationships that could be construed as a potential conflict of interest.

## Publisher’s note

All claims expressed in this article are solely those of the authors and do not necessarily represent those of their affiliated organizations, or those of the publisher, the editors and the reviewers. Any product that may be evaluated in this article, or claim that may be made by its manufacturer, is not guaranteed or endorsed by the publisher.
